# Health and Performance in the National Para Powerlifting Team: Associations Between Injuries, Sleep Parameters, Nutritional Factors, Mood States, and Performance

**DOI:** 10.3390/ijerph23040459

**Published:** 2026-04-03

**Authors:** Thaiany de Paula Giacomini, Fabrizio Veloso Rodrigues, Thiago Fernando Lourenço, Samuel Bento da Silva, Vivian De Oliveira, Andre Luis Aroni

**Affiliations:** 1 Health Data Science Postgraduate Programs, São Francisco University (USF), Bragança Paulista 12916-900, Brazil; thaiany.giacomini@mail.usf.edu.br; 2 Brazilian Paralympic Committee, Sao Paulo 04329-000, Brazil; fabrizio.rodrigues@cpb.org.br (F.V.R.); thiago.lourenco@cpb.org.br (T.F.L.); samuel.silva@cpb.org.br (S.B.d.S.); 3 Faculty of Human Kinetics, University of Lisbon, 1499-002 Lisbon, Portugal; vivian_oliveira58@hotmail.com

**Keywords:** Paralympic athletes, health monitoring, sleep duration, mood states, sports-related injuries, athletic performance

## Abstract

**Highlights:**

**Public health relevance—How does this work relate to a public health issue?**

- Sleep patterns and mood states are modifiable health-related behaviors that directly affect physical and psycho-emotional states in athletes with disabilities.
- Monitoring health variables in Paralympic athletes addresses health equity by generating evidence for an underserved and underrepresented population in public health research.

**Public health significance—Why is this work of significance to public health?**

- The study provides empirical evidence linking sleep stability and positive mood states to performance-related outcomes in athletes with disabilities, expanding the public health knowledge base.
- Findings contribute to the development of preventive, health-oriented strategies aimed at supporting emotional state in elite disability sport.

**Public health implications—What are the key implications or messages for practitioners, policy makers and/or researchers in public health?**

- Public health practitioners should incorporate sleep and mood monitoring into health promotion programs targeting athletes with disabilities.
- Policy makers and researchers are encouraged to invest in longitudinal surveillance systems and inclusive research agendas to improve health outcomes in Paralympic and disability sport contexts.

**Abstract:**

**Background:** Monitoring health-related variables across a competitive season is essential to understand factors associated with performance in Paralympic athletes. However, evidence on the interplay between sleep, mood states, nutritional factors, injuries, and performance remains limited. **Objective:** To examine the associations between injuries, sleep parameters, nutritional factors, mood states, and performance in Para powerlifting athletes during a competitive cycle. **Methods:** Twenty-four athletes from the Brazilian National Para powerlifting team were assessed at three time points: baseline (~3 months pre-competition), pre-competition (upon arrival), and post-competition (day after the event). Data were collected using standardized instruments and analyzed in R. Descriptive statistics, Mann–Whitney U tests, Spearman’s correlations, Friedman tests, and individual delta values (Δ) were applied. **Results:** No significant between-group differences were observed in pre-competition cross-sectional analyses. Longitudinally, sleep duration was the only variable consistently differing between performance groups. Athletes who matched or improved performance showed greater sleep stability, whereas those who did not improve exhibited larger post-competition increases in sleep duration. Negative mood states decreased over time, and baseline vigor was higher in the higher-performing group. Sleep duration changes were negatively correlated with performance variation (ρ = −0.575, *p* = 0.003). **Conclusions:** Sleep duration was the variable most consistently associated with performance variation. Mood changes reflected reduced negative affect over time. Findings support longitudinal monitoring in Para powerlifting, although caution is warranted due to the observational design and small sample.

## 1. Introduction

Para powerlifting has evolved into a highly competitive Paralympic sport that demands maximal strength, technical precision, and rigorous training routines. Beyond performance outcomes, however, the health of para-athletes represents an important public health concern. Participation in sport among people with disabilities has been consistently associated with improvements in physical health, social inclusion, and overall quality of life, as highlighted in the World Health Organization report on disability and subsequent research on the Paralympic movement [1,2]. In addition, prospective epidemiological data, such as the 52-week cohort study conducted by Fagher et al. [3], demonstrate that health monitoring in Paralympic athletes is essential due to the considerable incidence of injuries and illnesses across a competitive season.

Despite these benefits, elite para-athletes are exposed to unique and multifactorial health risks. These risks emerge from the interaction between impairment-related conditions and sport-specific demands, including high training loads, travel-related stress, and environmental constraints associated with international competitions [4,5]. Furthermore, the classification systems that underpin Paralympic sport, although essential for fair competition, also reflect the heterogeneity of impairments and functional capacities, which may influence both training responses and health outcomes [6]. Within this context, understanding how these factors interact is critical for promoting both performance and long-term athlete health.

In this complex scenario, injury occurrence becomes a central determinant of athlete health and competitive availability. From a public health perspective, sports injuries are associated with functional limitations, increased healthcare utilization, and reduced quality of life [7]. This issue may be further exacerbated in para-athletes, who often experience elevated injury risk due to biomechanical compensations, asymmetrical loading patterns, and impairment-specific vulnerabilities, as discussed in the literature on Paralympic sports medicine [8]. In Para powerlifting specifically, the repetitive nature of upper limb loading and the high demands placed on trunk stabilization may increase susceptibility to overuse injuries, potentially compromising both performance and long-term musculoskeletal health.

In parallel with injury risk, sleep has emerged as a key factor influencing both health and athletic performance. Sleep quantity and quality are fundamental for physiological recovery, cognitive function, and emotional regulation. Evidence from clinical and epidemiological studies indicates that insufficient sleep duration is associated with increased risk of cardiometabolic disorders, impaired immune function, and poorer general health outcomes [9,10]. Within elite sport, sleep disturbances have been linked to decreased strength output, slower reaction times, impaired decision-making, and a higher likelihood of injury [11]. Importantly, para-athletes may be particularly vulnerable to sleep disruption due to factors such as chronic pain, spasticity, thermoregulatory challenges, medication use, and nighttime care routines [12], further highlighting the need for integrated health monitoring approaches in this population.

In addition to sleep and injury-related factors, nutritional status represents a fundamental determinant of both health and athletic performance. Adequate energy availability and nutrient intake are essential for maintaining immune function, supporting musculoskeletal integrity, and reducing the risk of fatigue-related injuries, as highlighted in the position statement by Thomas et al. [13], which synthesizes evidence-based recommendations for optimizing performance through nutrition. More recently, emerging evidence has highlighted the role of targeted nutritional strategies in mitigating muscle deficits and enhancing recovery following injury. For example, Smith et al. [14], in a comprehensive review of nutritional interventions after anterior cruciate ligament (ACL) injury and reconstruction, emphasize the importance of adequate protein intake, amino acid supplementation (particularly leucine), and creatine to attenuate quadriceps muscle atrophy and promote muscle protein synthesis during periods of reduced loading. The authors also discuss the potential role of omega-3 fatty acids and vitamin D in modulating inflammation and supporting neuromuscular recovery. Collectively, these findings reinforce the close relationship between nutrition, rehabilitation, and performance outcomes, particularly in contexts involving injury and return to sport.

However, translating these recommendations into practice may be particularly challenging for para-athletes. According to Fagher et al. [15], Paralympic athletes often present distinct physiological and practical considerations, including altered energy expenditure associated with different impairment types, gastrointestinal dysfunction, and logistical barriers to accessing specialized dietary support—especially during international competitions and travel. These constraints may compromise the ability to meet nutritional requirements consistently. Therefore, while nutrition constitutes a central pillar of athlete health, its interaction with other factors such as sleep and psycho-emotional states further emphasizes the importance of integrated and context-specific monitoring strategies.

Extending this multidimensional perspective, mood states represent another relevant component linking health and performance in sport. In the present study, mood was approached as a set of transient psycho-emotional states, rather than as a broader construct of mental health or clinical psychological functioning. This distinction is important because mood states may fluctuate in response to training load, travel demands, competition stress, and recovery conditions, making them particularly relevant for short-term athlete monitoring in applied sport settings. Within this perspective, the Profile of Mood States (POMS) has been widely used in sport science to assess dimensions such as tension, depression, anger–hostility, fatigue, confusion, and vigor, providing a practical framework for examining mood-related responses to training and competition [16]. A meta-analysis confirmed the widespread and valid use of the POMS in assessing mood states related to sport performance [17].

This framework was considered more appropriate for the present study than broader models centered on mental disorders, psychopathology, or general psychological well-being, because our objective was not to diagnose clinical conditions, but to monitor short-term mood variations potentially associated with performance across a competitive cycle. In this context, mood-state monitoring may help identify patterns related to recovery, fatigue, and competition readiness. In addition, sleep and mood are closely interconnected processes, since insufficient sleep may contribute to emotional dysregulation and fatigue, whereas negative mood states may also impair recovery and perceived readiness in athletes [18]. Thus, monitoring mood states alongside sleep and other health-related variables may offer a practical and context-sensitive approach for understanding performance-related fluctuations in elite Para powerlifting.

Despite growing recognition of the multidimensional determinants of athlete health, relatively few studies have examined the combined influence of injuries, sleep parameters, nutritional factors, and mood states on performance—particularly within the context of elite Para powerlifting. Most available evidence has addressed these variables in isolation, limiting a comprehensive understanding of their interactions. Importantly, several of these factors—such as sleep behaviors, dietary patterns, and psycho-emotional state—are potentially modifiable and carry broader public health implications. For instance, research by Cruz et al. [19] highlights the association between sleep quality and quality of life in Paralympic athletes, reinforcing the relevance of these variables beyond performance outcomes. Collectively, these considerations underscore the need for integrated, longitudinal approaches to monitoring health and performance in para-athlete populations.

Therefore, the aim of this study was to examine the associations between injuries, sleep parameters, nutritional factors, mood states and performance in Para powerlifting athletes during a competitive cycle. By adopting a holistic perspective, this research seeks to contribute to the understanding of how these factors relate to competitive performance in elite Para powerlifting.

## 2. Materials and Methods

### 2.1. Participants and Procedure

The study included 24 adult Brazilian Para powerlifting athletes of both sexes who were monitored throughout a competitive cycle. All participants were members of the Brazilian National Para Powerlifting team and completed assessments at three predefined time points: baseline, pre-competition, and post-competition.

Given the elite nature of the population, an intentional convenience sample was used, consisting of athletes who were officially competing during the monitored cycle and were available to complete all study procedures. Athletes were eligible for inclusion if they were actively participating in Para powerlifting competition during the study period and completed all assessments across the three time points.

Data collection was conducted at three standardized moments: (1) baseline, in Brazil, approximately three months before the target competition; (2) pre-competition, upon arrival at the competition venue and before the start of official activities; and (3) post-competition, on the day after each athlete’s competitive event. All participants completed the full monitoring protocol at all three moments.

This study was conducted in accordance with the Declaration of Helsinki and was approved by the Research Ethics Committee of the São Francisco University (CAAE: 85293224.8.0000.5514). All participants were informed about the study procedures and provided written informed consent prior to participation.

### 2.2. Experimental Design

The monitoring period was standardized for all participants and began approximately three months before the target competition, during the preparatory phase (baseline). All athletes subsequently competed in the same international event, the World Para Powerlifting Championship, held in Cairo, Egypt. This shared competitive context provided a common setting in terms of travel demands, competition routine, and environmental exposure. In addition, the competition site involved an approximately 6 h time difference relative to Brazil, which was considered when interpreting sleep-related variables.

Data were collected at three standardized time points for all athletes: (1) baseline, in Brazil, approximately three months before the competition, during the preparatory period; (2) pre-competition, upon arrival in Cairo and before the start of official competition activities; and (3) post-competition, on the day after each athlete’s event. This design enabled longitudinal comparisons across the preparatory phase, the travel/acclimatization period, and the immediate post-competition period.

All questionnaires were administered digitally through a web-based platform and completed by the athletes using personal electronic devices. Standardized instructions were provided by the research team at all assessment moments. One of the authors accompanied the team during the competition period and supervised questionnaire administration on site, which helped standardize data collection procedures and monitor completion of the assessments across participants and time points.

### 2.3. Measures

Health problems were monitored using the Oslo Sports Trauma Research Center Questionnaire on Health Problems (OSTRC-H2), validated for Brazilian Portuguese [20]. This instrument assesses the impact of injuries and illnesses on sports participation, training volume, sports performance, and symptom severity. Total severity scores range from 0 to 100, with higher values indicating a greater health burden. The Brazilian version has demonstrated satisfactory internal consistency, with Cronbach’s α values above 0.80.

Dietary patterns were assessed using a Food Frequency Questionnaire (FFQ) adapted for the Brazilian population [21]. The FFQ was used to characterize habitual food consumption across major food groups and selected dietary items over the reference period. For the present study, an FFQ-derived score was used as a summary indicator of dietary pattern at each assessment time point. The instrument has shown adequate validity and reproducibility in Brazilian epidemiological studies.

Mood states were evaluated using the Profile of Mood States (POMS), validated in Portuguese [22]. The questionnaire includes the dimensions tension, depression, anger–hostility, fatigue, confusion, and vigor. In addition to the subscale scores, a Total Mood Disturbance (TMD) score was calculated by summing the negative mood dimensions and subtracting vigor, according to the instrument’s standard scoring approach. Higher TMD values indicate greater overall mood disturbance. The Brazilian version has demonstrated good reliability, with Cronbach’s α ranging from 0.70 to 0.90 across subscales.

Daytime sleepiness was assessed using the Epworth Sleepiness Scale (ESS), validated in Portuguese [23]. The ESS consists of eight items scored from 0 to 3, generating a total score ranging from 0 to 24. Scores above 10 indicate excessive daytime sleepiness. The ESS has shown adequate reliability and validity across different populations.

Sleep was assessed using the Pittsburgh Sleep Quality Index (PSQI), validated for use in Brazil [24]. The PSQI evaluates sleep quality over the previous month across seven components and generates a global score ranging from 0 to 21, with scores above 5 indicating poor sleep quality. In addition to the global PSQI score, self-reported sleep duration (hours) was analyzed as a separate continuous variable at each assessment time point. The Brazilian version of the PSQI has demonstrated satisfactory reliability, with Cronbach’s α of approximately 0.80. Body mass was included as an anthropometric variable and analyzed at baseline, pre-competition, and post-competition.

### 2.4. Data Analysis

Initially, all variables were inspected for missing data, distributional characteristics, and potential inconsistencies. Normality was assessed separately by performance group and assessment time point using the Shapiro–Wilk test, combined with visual inspection of histograms, quantile-quantile plots, and boxplots. Given the small sample size and the recurrent violation of normality assumptions, nonparametric procedures were adopted for the main inferential analyses [25,26].

Continuous variables are presented as median and interquartile range (IQR), whereas categorical variables are described using absolute and relative frequencies. Sample characterization was based exclusively on baseline data.

For group-based analyses, athletes were classified according to their performance outcome at the World Championship. The YES group included athletes whose World Championship result was equal to or greater than their previous season-best performance, whereas the NO group included athletes whose World Championship result was lower than their previous season-best performance. Cross-sectional comparisons between groups in the pre-competition period were performed using the Mann–Whitney U test for injury indicators, sleep parameters, mood-related variables, dietary score, and body mass [27]. Effect sizes were calculated using the r coefficient, obtained by dividing the standardized Z statistic by the square root of the total sample size (r = Z/√N). Effect sizes were interpreted according to conventional thresholds, with values of approximately 0.1, 0.3, and 0.5 representing small, moderate, and large effects, respectively [28,29].

For longitudinal analyses, individual change scores were calculated relative to baseline as follows: ΔPre-Baseline = pre-competition value − baseline value and ΔPost-Baseline = post-competition value − baseline value. This approach was adopted to reduce the influence of interindividual variability and to allow comparisons of changes over the competitive cycle rather than comparisons based solely on absolute values [30]. Between-group comparisons for both ΔPre-Baseline and ΔPost-Baseline were also performed using the Mann–Whitney U test, with corresponding effect sizes (r) calculated.

To investigate associations between longitudinal changes and sports performance, Spearman’s rank correlations (ρ) were calculated using the ΔPost-Baseline values of the monitored variables. This method was selected because it is appropriate for small samples, does not require normally distributed data, and allows the assessment of monotonic associations between continuous or ordinal variables [31]. Performance variation was defined as the difference between the World Championship result and the athlete’s previous season-best performance (ΔPerformance = World Championship result−previous season-best performance). Thus, positive values indicated that the athlete matched or exceeded the previous season-best result, whereas negative values indicated lower performance at the World Championship relative to the prior season’s best. Correlations were also examined in relation to final ranking. Given the exploratory nature of the study, emphasis was placed on the magnitude and direction of the correlation coefficients, and *p*-values were interpreted cautiously.

All statistical analyses were performed using R software (R Foundation for Statistical Computing, Vienna, Austria). Statistical significance was set at *p* < 0.05. Because of the exploratory nature of the study and the small sample size, no formal correction for multiple comparisons was applied. Therefore, results were interpreted conservatively, with emphasis on effect sizes, consistency across analytical approaches, and the overall plausibility of the observed findings [32].

## 3. Results

### 3.1. Sample Characteristics

The sample comprised 24 Brazilian Para powerlifting athletes monitored across the competitive cycle. Baseline characteristics of the sample are presented in Table 1 and Table 2. Continuous baseline variables included body mass, sleep-related indicators, health-problem burden, mood state, and dietary score.

At baseline, the median sleep duration was 7.75 h, the median PSQI global score was 4.00, and the median ESS score was 8.50. The median OSTRC-H2 severity score was 0.00, although variability was observed across participants. Median body mass was 70.5 kg, median TMD score was 114.0, and the median FFQ-derived dietary score was 30.5.

In addition to the continuous variables, the sample was characterized according to sex, educational level, monthly income, marital status, presence of children, and impairment origin, as shown in Table 2.

Overall, the sample showed heterogeneity in both educational attainment and socioeconomic background, as well as in impairment origin. These descriptive data provide contextual information for the interpretation of the longitudinal analyses presented in the subsequent sections.

### 3.2. Cross-Sectional Comparisons in the Pre-Competition Period

Cross-sectional comparisons performed during the pre-competition period between the YES group (athletes who matched or improved their previous season-best performance at the World Championship; *n* = 16) and the NO group (athletes who did not match or improve their previous season-best performance; *n* = 8) revealed no statistically significant differences for any of the analyzed variables.

Regarding sleep-related variables, no between-group differences were observed for sleep duration (YES: 8.00 h [IQR: 1.12] vs. NO: 8.00 h [IQR: 1.62]; *p* = 0.98), sleep quality score (YES: 5.00 [IQR: 4.00] vs. NO: 3.00 [IQR: 2.50]; *p* = 0.66), or daytime sleepiness (YES: 8.50 [IQR: 7.25] vs. NO: 4.00 [IQR: 8.50]; *p* = 0.30). Although not statistically significant, daytime sleepiness showed the largest effect size among the analyzed variables (r = 0.22).

No significant between-group differences were found for injury burden, with both groups presenting median OSTRC-H2 scores of 0.00 (YES: [IQR: 0.00] vs. NO: [IQR: 0.00]; *p* = 0.54). Similarly, TMD did not differ between groups (YES: 101.00 [IQR: 17.00] vs. NO: 102.00 [IQR: 16.00]; *p* = 0.74).

No statistically significant differences were observed for the FFQ-derived dietary score (YES: 31.70 [IQR: 19.30] vs. NO: 33.30 [IQR: 17.80]; *p* = 0.78) or body mass (YES: 65.00 kg [IQR: 31.38] vs. NO: 81.95 kg [IQR: 42.20]; *p* = 0.48).

Overall, effect sizes for pre-competition cross-sectional comparisons were classified as trivial to small (|r| ≤ 0.22), indicating no meaningful between-group differences at this stage of the competitive cycle.

### 3.3. Longitudinal Analyses Based on Delta Changes Across the Competitive Cycle

#### 3.3.1. Sleep Parameters

**Figure 1:**
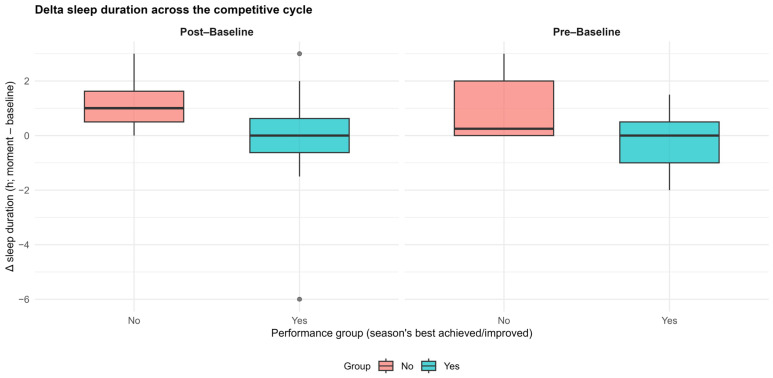
Changes in sleep duration across the competitive cycle. Boxplots showing delta (Δ) changes in sleep duration relative to baseline for the pre-competition (ΔPre-Baseline) and post-competition (ΔPost-Baseline) periods, stratified by performance group. The central line represents the median, boxes indicate the interquartile range (IQR), and whiskers represent the range excluding outliers. Athletes were classified according to whether they matched or improved their previous season-best performance at the World Championship.

Longitudinal analyses based on delta changes (Δ), calculated relative to baseline, showed statistically significant between-group differences only for sleep duration. Significant differences were observed for both ΔPre-Baseline (*p* = 0.049) and ΔPost-Baseline (*p* = 0.026), indicating distinct sleep-duration trajectories between performance groups across the competitive cycle (Figure 1).

For ΔPre-Baseline, the YES group showed relative stability in sleep duration (median: 0.00 h [IQR: −0.75 to 0.75]), whereas the NO group showed a small increase (median: 0.25 h [IQR: −0.75 to 1.25]). This difference was statistically significant and associated with a moderate effect size (r = −0.40).

A similar pattern was observed for ΔPost-Baseline. The YES group again maintained relative stability in sleep duration (median: 0.00 h [IQR: −0.75 to 0.50]), whereas the NO group showed a more pronounced increase (median: 1.00 h [IQR: 0.44 to 1.56]). This difference was statistically significant and showed a moderate-to-large effect size (r = −0.46).

In contrast, no statistically significant between-group differences were observed for changes in body mass in either the Pre-Baseline period (YES: 0.00 kg [IQR: −0.85 to 0.85] vs. NO: 0.00 kg [IQR: −1.10 to 1.10]; *p* = 0.924; r = −0.03) or the Post-Baseline period (YES: −0.20 kg [IQR: −0.70 to 0.30] vs. NO: 0.00 kg [IQR: −0.85 to 0.85]; *p* = 0.592; r = −0.11).

Likewise, no significant between-group differences were identified for the FFQ-derived dietary score in the Pre-Baseline period (YES: 1.10 [IQR: −1.63 to 3.83] vs. NO: 3.10 [IQR: −2.95 to 9.15]; *p* = 0.878; r = −0.04) or the Post-Baseline period (YES: −0.20 [IQR: −6.35 to 5.95] vs. NO: −7.30 [IQR: −12.85 to −1.75]; *p* = 0.312; r = 0.21).

For injury burden, OSTRC-H2 scores were centered at zero in both groups in both comparison periods, with no statistically significant differences (both *p* = 0.438; r = 0.14). Similarly, no significant between-group differences were observed for TMD, sleep quality score, or daytime sleepiness, with effect sizes predominantly classified as trivial to small (|r| ≤ 0.19).

Overall, among the monitored variables, only sleep duration showed consistent longitudinal differences between performance groups, with greater stability across the competitive cycle in the YES group and greater post-competition increases in the NO group.

#### 3.3.2. Injuries, Mood States, Nutritional Factors, and Body Mass

No statistically significant differences were observed between the YES and NO groups for delta changes in OSTRC-H2 score, TMD, FFQ-derived dietary score, or body mass, for either ΔPre-Baseline or ΔPost-Baseline (all *p* > 0.31). Effect sizes for these comparisons were classified as trivial to small, indicating no consistent between-group differences in the longitudinal trajectories of these variables across the competitive cycle.

However, an exploratory baseline comparison of POMS dimensions revealed a significantly higher baseline vigor score in the YES group than in the NO group (*p* = 0.034).

### 3.4. Associations Between Longitudinal Changes (Δ) and Sports Performance

Spearman rank correlation analyses were performed to examine the associations between ΔPost-Baseline changes in the monitored variables and two performance outcomes: performance variation and final ranking, as presented in Table 3. Performance variation was defined as the World Championship result minus the athlete’s previous season’s best performance.

A moderate-to-strong negative correlation was observed between changes in sleep duration and performance variation (ρ = −0.575; *p* = 0.003; *n* = 24). Considering the direction of the performance-variation score, this result indicates that greater increases in sleep duration from baseline to post-competition were associated with lower values of ΔPerformance, whereas better relative performance was more closely associated with greater stability in sleep duration across the competitive cycle.

No statistically significant associations were found between performance variation and changes in body mass, injury burden, TMD, dietary score, daytime sleepiness, or sleep quality. Likewise, no statistically significant associations were observed between final ranking and longitudinal changes in any of the analyzed variables.

Overall, among the monitored variables, only changes in sleep duration showed a statistically significant association with continuous performance variation, whereas no meaningful associations were observed for the remaining variables or for final ranking.

### 3.5. Mood State Trajectory Across the Competitive Cycle

Longitudinal analyses of mood states across the competitive cycle identified changes in several POMS dimensions, as observed both in individual delta values (Δ) relative to baseline and in the overall comparisons across the three assessment time points (baseline, pre-competition, and post-competition).

#### 3.5.1. Individual Changes (Δ) Relative to Baseline

Longitudinal analyses of individual delta values relative to baseline showed changes in several POMS dimensions across the competitive cycle (Table 4). Overall, negative mood dimensions tended to decrease over time.

For depression, median values decreased already in the pre-competition period (ΔPre-Baseline: −1.5 [IQR: −6.0 to 0.0]) and decreased further in the post-competition period (ΔPost-Baseline: −3.5 [IQR: −6.0 to −0.8]). A similar pattern was observed for anger–hostility (ΔPre-Baseline: −1.0 [IQR: −4.2 to 0.0]; ΔPost-Baseline: −2.5 [IQR: −6.0 to 0.0]) and fatigue (ΔPre-Baseline: −3.0 [IQR: −4.2 to 0.0]; ΔPost-Baseline: −1.5 [IQR: −6.0 to 0.2]).

The TMD score also decreased relative to baseline in both the pre-competition period (median: −10.0 [IQR: −24.2 to 0.0]) and the post-competition period (median: −13.0 [IQR: −24.0 to −4.0]). In contrast, vigor remained relatively stable in the pre-competition period and was higher in the post-competition assessment (ΔPre-Baseline: 0.0 [IQR: −3.2 to 5.5]; ΔPost-Baseline: 3.0 [IQR: −4.0 to 6.0]) (Table 4).

#### 3.5.2. Global Comparisons Across Baseline, Pre-Competition, and Post-Competition

Global comparisons across the three assessment time points showed significant changes in several POMS dimensions across the competitive cycle (Table 5). Friedman test results indicated significant differences for depression (χ^2^ = 15.68; *p* = 0.000394), anger–hostility (χ^2^ = 12.17; *p* = 0.00228), fatigue (χ^2^ = 8.55; *p* = 0.0139), confusion (χ^2^ = 6.49; *p* = 0.039), and total mood disturbance (TMD) (χ^2^ = 11.76; *p* = 0.00279).

Across these dimensions, scores generally decreased from baseline to pre-competition and remained below baseline levels in the post-competition assessment, although slight increases were observed in some variables after the event. In contrast, vigor did not change significantly across time (χ^2^ = 0.35; *p* = 0.84), whereas tension showed a non-significant trend toward change (χ^2^ = 5.74; *p* = 0.0566) (Table 5).

### 3.6. Associations Between Mood State Changes and Competitive Performance

Associations between changes in POMS scores across the competitive cycle (ΔPOMS = post-competition−baseline) and continuous indicators of sports performance were examined using Spearman rank correlations.

#### 3.6.1. ΔPOMS and Final Event Ranking

No statistically significant associations were observed between changes in POMS dimensions and final event ranking. Correlation coefficients ranged from trivial to small magnitudes (|ρ| ≤ 0.27). The largest coefficient was observed for anger–hostility, which showed a small negative correlation with final ranking (ρ = −0.27; *p* = 0.196), indicating no consistent relationship between changes in this dimension and competitive ranking.

The remaining dimensions also showed no significant associations with final ranking: vigor (ρ = 0.01; *p* = 0.959), tension (ρ = 0.08; *p* = 0.722), depression (ρ = −0.14; *p* = 0.529), fatigue (ρ = −0.12; *p* = 0.586), confusion (ρ = −0.06; *p* = 0.768), and total mood disturbance (TMD) (ρ = −0.16; *p* = 0.457).

#### 3.6.2. ΔPOMS and Performance Variation (ΔPerformance)

No statistically significant associations were identified between changes in POMS scores and performance variation, defined as the difference between the World Championship result and the athlete’s previous season-best performance. Most correlations were trivial in magnitude (|ρ| < 0.20).

The largest coefficient was observed for vigor, which showed a small-to-moderate negative correlation with ΔPerformance (ρ = −0.39; *p* = 0.062). Although this result did not reach statistical significance, it may suggest a possible trend; however, this finding should be interpreted cautiously.

## 4. Discussion

This study advances current knowledge by demonstrating that the longitudinal consistency of sleep patterns, rather than sleep volume alone, together with mood state trajectories, is associated with performance improvements in elite Para powerlifting athletes. While previous research has established sleep as a determinant of athletic performance [10,33], the present findings extend this evidence to Para powerlifting, a population still underrepresented in the literature.

Sleep stability emerged as an important factor associated with improved performance. Athletes who improved their results maintained more consistent sleep patterns across the competitive cycle, supporting evidence that sleep restriction impairs neuromuscular performance, cognitive function, and recovery processes [33]. However, much of this literature is based on able-bodied athletes, limiting its direct applicability to Paralympic power sports. In this context, the present findings suggest that sleep may play an even more critical role in Para powerlifting, where maximal strength and recovery from high mechanical loads are decisive. This is consistent with expert consensus highlighting sleep as a cornerstone of athlete health and performance, emphasizing not only sleep quantity but also consistency and individualized monitoring strategies [34].

Despite these recommendations, implementation remains inconsistent, particularly in Paralympic sport. Evidence from athletes with disabilities indicates that psychological factors such as pre-competition stress and emotional states may interact with recovery processes and influence performance outcomes. For example, Koper et al. [35], in a study of elite boccia athletes with disabilities, demonstrated that pre-competition emotional states—including levels of anxiety, emotional tension, and readiness—were significantly associated with subsequent performance results, highlighting the impact of psychological readiness on competitive outcomes. These findings reinforce the need for integrated monitoring approaches that consider both physiological and psychological dimensions of athlete health.

From a public health perspective, these findings reinforce the concept of sleep health as a multidimensional and modifiable determinant [36]. Despite growing recognition, sleep remains insufficiently addressed in athlete health surveillance systems, which traditionally prioritize injury and illness monitoring [3,8]. The present data support expanding these systems to include sleep metrics, especially given evidence linking sleep disturbances to increased injury risk, impaired immune function, and health burden [37]. However, in this case, the results did not correlate sleep deprivation with injuries in these Para powerlifting athletes.

Mood state trajectories further differentiate performance outcomes. Higher baseline vigor and reductions in depression, anger–hostility, fatigue, and confusion suggest improved psycho-emotional state among athletes who enhanced performance. This profile aligns with the classic “iceberg profile” associated with optimal performance. However, its predictive validity has been debated in heterogeneous athletic populations, such as the study by Rice and colleagues [38]. The current findings provide longitudinal support for mood monitoring in Paralympic strength sports, although caution is warranted when generalizing across contexts. Elite athletes experience unique psychosocial stressors, and Paralympic athletes may face additional challenges related to disability, classification, and accessibility, which are often overlooked in traditional frameworks [2,5,6,35].

Notably, the global reduction in negative mood states across the competitive cycle contrasts with literature describing increased psychological strain during intensified training phases [38]. Given the established reliability and widespread use of the POMS for monitoring mood in athletic populations [17], this discrepancy may reflect effective periodization and support structures within this national team, highlighting the protective role of well-designed training environments. Nevertheless, this interpretation is not uniformly supported, and the literature remains limited regarding the association between reductions in negative mood states, as assessed by the Profile of Mood States (POMS), and superior athletic performance, particularly among Para-athletes. As illustrated by surveillance data from the Rio 2016 Paralympic Games, which reported substantial illness and injury rates, approximately 10 cases per 1000 athlete-days were reported and more than 12% of athletes were affected during the Games [39], underscoring that health risks remain significant and that psychological and sleep factors should be integrated into comprehensive monitoring models. Importantly, the Paralympic context remains underrepresented in the literature, warranting further research to reduce biases derived from mainstream sport [5,40].

Taking a broader perspective on the findings, recent evidence reinforces that sleep disturbances are particularly pronounced in Paralympic competition settings due to travel demands, classification schedules, environmental barriers, and accessibility constraints. A narrative review of sleep disruption in Paralympic athletes at the Tokyo 2021 Games highlighted that congested competition timetables, unfamiliar sleep environments, and heightened pre-competition anxiety contributed to reduced sleep quality and duration [41]. Importantly, these challenges were further exacerbated by the context of the COVID-19 pandemic, which introduced additional stressors such as strict health protocols, social isolation, uncertainty regarding competition schedules, and limited access to usual support systems. These factors may have intensified emotional strain, mood disturbances, and anxiety levels, thereby further compromising sleep patterns and recovery processes in Paralympic athletes.

These findings align with the present results and can be interpreted through the lens of Self-Regulation Theory in sport. According to Toering et al. [42], self-regulation encompasses athletes’ ability to plan, monitor, evaluate, and adapt their behaviors, thoughts, and emotional responses to achieve performance goals under varying conditions. This process involves key components such as goal setting, self-monitoring, effort regulation, and reflective evaluation, which are particularly relevant in high-performance contexts characterized by stress and uncertainty. Within this framework, maintaining stable sleep patterns and mood states may represent an important behavioral strategy that supports effective self-regulation, enabling athletes to better manage training demands, emotional states, and recovery processes.

In this context, sleep emerges as a critical self-regulatory resource, and its disruption may compromise athletes’ capacity to adapt to competitive demands, particularly among Paralympic athletes facing additional logistical and disability-related challenges (NO group case). Although previous studies have highlighted the impact of travel-related constraints on sleep disturbances, the present study adopts a longitudinal approach to specifically investigate sleep stability across the competitive cycle, providing a more comprehensive perspective on sleep behavior over time.

Environmental conditions represent an additional and often underestimated determinant of sleep health in elite sport. Although environmental temperature was not directly controlled in the present study, external factors may pose challenges to sleep stability in para-athletes. Against this backdrop, projections for the Paris 2024 Olympic and Paralympic Games have indicated that elevated nighttime temperatures can negatively affect sleep onset, efficiency, and recovery processes, particularly under conditions of heat stress and insufficient cooling [43]. These environmental pressures may disproportionately affect Paralympic athletes due to altered thermoregulation, medication use, or limited access to adaptive cooling strategies.

Within the framework of the Integrated Model of Stress and Recovery, such environmental stressors may accumulate alongside training load and travel fatigue, disrupting the dynamic balance between stress and recovery that underpins optimal performance and health. Kellmann [44] emphasizes that recovery is a multidimensional process involving physiological, psychological, and behavioral components, and that inadequate recovery relative to accumulated stress can lead to maladaptation, performance decrements, and increased risk of overtraining. In this perspective, insufficient or unstable sleep may act as a critical mediator, impairing the restoration processes required to maintain this balance over time. The present findings reinforce that maintaining sleep stability—even in the presence of environmental and logistical stressors—may serve as a protective factor within this dynamic stress–recovery balance, while also being accompanied by reductions in negative mood dimensions, suggesting improved psycho-emotional regulation across the competitive cycle.

Finally, emerging evidence indicates that sleep disturbances in Paralympic athletes are associated not only with performance decrements but also with increased susceptibility to injuries and illnesses [45]. Although the present study did not observe a direct association between sleep deprivation and injuries, the broader literature suggests that cumulative sleep deficits may impair immune function, delay tissue repair, and increase musculoskeletal injury risk over time. In particular, experimental and epidemiological evidence demonstrates that sleep restriction negatively affects psychomotor vigilance, reaction time, and cognitive processing speed—key determinants of sport performance, as presented by Walker [46]. Moreover, in a study with young athletes, sleeping less than 8 h per night has been shown to present a significantly higher risk of injury, likely due to impaired recovery and altered neuromuscular control [47].

From a theoretical standpoint, both the Self-Regulation Theory in Sport and the Integrated Model of Stress and Recovery support the integration of sleep monitoring into comprehensive, disability-informed health frameworks, as sleep influences emotional regulation, physiological recovery, and adaptive capacity under stress [42,44]. Together, these findings strengthen the argument that sleep health and mood states should be embedded within multi-dimensional public health strategies aimed at safeguarding performance and reducing health risks in Paralympic sport.

The present findings challenge the narrow biomedical focus of traditional athlete health models by highlighting sleep and psycho-emotional state as central determinants of both performance and overall health. Nevertheless, the results should be interpreted considering limitations inherent to longitudinal designs, including the small sample drawn from a single national team and the reliance on self-reported measures, which may be subject to reporting bias over repeated assessments. Additionally, potential floor effects in some questionnaire responses (e.g., OSTRC-H2 and FFQ) may have reduced the sensitivity of the instruments to detect meaningful within- and between-subject variability over time.

Although the longitudinal approach strengthens the ability to explore temporal relationships, residual confounding cannot be ruled out, as relevant contextual variables—such as fluctuations in training load, travel demands, environmental conditions, and recovery strategies—may not have been fully captured across all time points. Future research should aim to refine longitudinal monitoring frameworks, establish clearer causal pathways between sleep variability and performance, and incorporate more comprehensive and objective measures within integrated surveillance systems. Overall, from a public health perspective, prioritizing sleep health and psycho-emotional monitoring may contribute not only to performance optimization but also to health protection.

## 5. Conclusions

In summary, this study examined health-related factors associated with performance in a national Para powerlifting team, suggesting that sleep stability and balanced mood states may contribute to competitive success. Athletes who improved their performance in the World competition reported more stable sleep patterns and reductions in negative mood state dimensions. These findings support the role of sleep as a modifiable behavior that underpins recovery, emotional regulation, and performance readiness. From a public health perspective, and considering the presented limitations, promoting stable and adequate sleep among elite Para athletes may enhance performance while supporting improved mood regulation. Furthermore, routine monitoring of sleep and mood may provide a practical and low-cost approach for the early identification of excessive fatigue, maladaptation to training or environmental demands (e.g., international competitions), and potential declines in health and performance. Future research should further explore these relationships using larger samples and multimodal assessments to better understand the dynamic interplay between sleep, mood and performance in Paralympic sport.

## Figures and Tables

**Table 1:** Baseline characteristics of the sample (continuous variables).

Variable	Median	IQR	Min	Max
PSQI global score	4.0	3.25	1.0	9.0
ESS score	8.5	9.0	0.0	23.0
Body mass (kg)	70.5	36.08	40.6	133.0
Total mood disturbance (TMD) score	114.0	12.0	79.0	148.0
FFQ-derived dietary score	30.5	15.75	12.0	66.0
OSTRC-H2 severity score	0.0	18.0	0.0	50.0
Sleep duration (h)	7.75	2.0	6.0	10.5

IQR, interquartile range.

**Table 2:** Baseline characteristics of the sample (categorical variables).

Variable	Category	*n*	%
Sex	Male	9	37.5%
	Female	15	62.5%
Education level	Primary education	6	25.0%
	Secondary education	12	50.0%
	University degree	6	25.0%
Monthly income	Up to BRL 4.999	8	33.3%
	BRL 5.000–9.999	4	16.7%
	BRL 10.000–14.999	4	16.7%
	Above BRL 15.000	4	16,7%
	Not informed	4	16.7%
Marital status	Single	20	83.3%
	Married	4	16.7%
Children	0	17	70.8%
	1	5	20.8%
	2	2	8.3%
Impairment origin	Congenital	19	79.2%
	Acquired	5	20.8%

Percentages are based on the total sample (*n* = 24).

**Table 3:** Spearman’s rank correlations (ρ) between longitudinal changes (ΔPost-Baseline) in monitored variables and performance outcomes.

Outcome	Predictor	ρ	*p*-Value
ΔPerformance (World − season best)	Δ sleep duration (post − baseline, h)	−0.575	0.00329
ΔPerformance (World − season best)	Δ body mass (post − baseline, kg)	0.202	0.343
ΔPerformance (World − season best)	Δ OSTRC-H2 severity score (post − baseline)	0.155	0.471
ΔPerformance (World − season best)	Δ total mood state score (post − baseline)	0.144	0.503
ΔPerformance (World − season best)	Δ FFQ-derived dietary score (post − baseline)	0.088	0.683
ΔPerformance (World − season best)	Δ sleepiness score (post − baseline)	0.068	0.754
ΔPerformance (World − season best)	Δ sleep quality score (post − baseline)	−0.055	0.797
Final ranking	Δ sleepiness score (post − baseline)	0.168	0.432
Final ranking	Δ sleep duration (post − baseline, h)	0.161	0.453
Final ranking	Δ OSTRC-H2 severity score (post − baseline)	−0.160	0.454
Final ranking	Δ total mood state score (post − baseline)	−0.159	0.457
Final ranking	Δ sleep quality score (post − baseline)	0.159	0.458
Final ranking	Δ FFQ-derived dietary score (post − baseline)	0.099	0.646
Final ranking	Δ body mass (post − baseline, kg)	0.084	0.695

Note. ΔPerformance = World Championship result − previous season-best performance. Positive values indicate that the athlete matched or exceeded the previous season-best result.

**Table 4:** Individual deltas (ΔPre-Baseline and ΔPost-Baseline) for POMS dimensions and total mood disturbance.

Variable	*n*	ΔPre-Baseline Median [IQR]	ΔPost-Baseline Median [IQR]
Vigor	24	0.0 [−3.2 to 5.5]	3.0 [−4.0 to 6.0]
Tension	24	−2.0 [−4.2 to 0.0]	−1.0 [−2.2 to 1.0]
Depression	24	−1.5 [−6.0 to 0.0]	−3.5 [−6.0 to −0.8]
Anger–hostility	24	−1.0 [−4.2 to 0.0]	−2.5 [−6.0 to 0.0]
Fatigue	24	−3.0 [−4.2 to 0.0]	−1.5 [−6.0 to 0.2]
Confusion	24	−1.0 [−3.2 to 0.0]	−0.5 [−3.2 to 1.2]
Total mood disturbance (TMD)	24	−10.0 [−24.2 to 0.0]	−13.0 [−24.0 to −4.0]

Note. IQR = interquartile range.

**Table 5:** Global comparisons of POMS dimensions across baseline, pre-competition, and post-competition.

Variable	Baseline Median [IQR]	Pre-Competition Median [IQR]	Post-Competition Median [IQR]	Friedman χ^2^	df	*p*-Value
Vigor	12.0 [8.0 to 16.2]	15.5 [8.5 to 18.2]	14.0 [9.2 to 18.2]	0.35	2	0.84
Tension	7.0 [6.0 to 9.0]	6.0 [2.8 to 7.2]	6.0 [4.0 to 9.0]	5.74	2	0.0566
Depression	4.5 [1.0 to 8.0]	1.0 [0.0 to 3.0]	0.0 [0.0 to 1.2]	15.68	2	0.000394
Anger–hostility	4.0 [1.0 to 6.2]	1.0 [0.0 to 4.0]	1.0 [0.0 to 2.0]	12.17	2	0.00228
Fatigue	6.0 [1.8 to 8.5]	2.0 [1.0 to 5.2]	2.0 [2.0 to 4.2]	8.55	2	0.0139
Confusion	6.0 [5.0 to 8.0]	5.0 [2.8 to 7.0]	6.0 [4.0 to 8.0]	6.49	2	0.039
Total mood disturbance (TMD)	114.0 [110.8 to 122.8]	101.0 [94.8 to 111.5]	104.5 [97.0 to 109.0]	11.76	2	0.00279

Note. IQR = interquartile range.

## Data Availability

The data that support the findings of this study are not publicly available due to privacy and ethical restrictions. Data may be available from the corresponding author upon reasonable request and with permission from the relevant ethics committee.

## Abbreviations

The following abbreviations are used in this manuscript:

FFQ	Food Frequency Questionnaire
OSTRC-H2	Oslo Sports Trauma Research Center Questionnaire on Health Problems
POMS	Profile Mood States
TMD	Total Mood Disturbance
ESS	Epworth Sleepiness Scale
PSQI	Pittsburgh Sleep Disturbance
IQR	Interquartile Range
